# Adiposity and grip strength as long-term predictors of objectively measured physical activity in 93 015 adults: the UK Biobank study

**DOI:** 10.1038/ijo.2017.122

**Published:** 2017-06-06

**Authors:** Y Kim, T White, K Wijndaele, S J Sharp, N J Wareham, S Brage

**Affiliations:** 1 MRC Epidemiology Unit, University of Cambridge School of Clinical Medicine, Institute of Metabolic Science, Cambridge Biomedical Campus, Cambridge, UK

## Abstract

**Background/Objectives:**

Fatness and fitness are associated with physical activity (PA) but less is known about the prospective associations of adiposity and muscle strength with PA. This study aimed to determine longitudinal associations of body mass index (BMI), waist circumference (WC) and grip strength (GS) with objectively measured PA.

**Subjects/Methods:**

Data are from the UK Biobank study. At baseline (2006–2010), BMI, WC and GS were objectively measured. At follow-up (2013–2015), a sub-sample of 93 015 participants (52 161 women) wore a tri-axial accelerometer on the dominant wrist for 7 days. Linear regression was performed to investigate longitudinal associations of standardised BMI, WC and GS at baseline with moderate-to-vigorous PA (MVPA) and acceleration after a median 5.7-years follow-up (interquartile range: 4.9–6.5 years).

**Results:**

Linear regression revealed strong inverse associations for BMI and WC, and positive associations for GS with follow-up PA; in women, MVPA ranges from lowest to highest quintiles of GS were 42–48 min day^−1^ in severely obese (BMI⩾35 kg m^−^^2^), 52–57 min day^−1^ in obese (30⩽BMI<35 kg m^−^^2^), 61–65 min day^−1^ in overweight (25⩽BMI<30 kg m^−^^2^) and 69–75 min day^−1^ in normal weight (18.5⩽BMI<25 kg m^−2^). Follow-up MVPA was also lower in the lowest GS quintile (42–69 min day^−1^) compared with the highest GS quintile (48–75 min day^−1^) across BMI categories in women. The pattern of these associations was generally consistent for men, and in analyses using WC and mean acceleration as exposure and outcome, respectively.

**Conclusions:**

More pronounced obesity and poor strength at baseline independently predict lower activity levels at follow-up. Interventions and policies should aim to improve body composition and muscle strength to promote active living.

## Introduction

Increasing physical activity (PA) levels is an important public health priority as physical inactivity is associated with increased risks of mortality as well as cardiovascular events.^1^ Studies^2, 3, 4^ have been performed to examine correlates and determinants of PA in order to inform interventions and policies aimed at increasing PA levels. Of various genetic, biological, behavioural and environmental factors influencing PA, adiposity was found to be inversely associated with PA in previous cross-sectional studies.^5, 6, 7, 8^ Some longitudinal studies reported that higher adiposity levels at baseline were associated with lower PA levels at follow-up.^9, 10, 11, 12^

In addition to adiposity, grip strength (GS), as a proxy measure for overall muscle strength,^13^ has been known to be a correlate of PA.^8, 14, 15^ The longer-term relationship between GS and self-report PA has been examined in one previous longitudinal study.^16^ In fact, all but one^10^ prior longitudinal studies^9, 11, 12, 16^ in this area of research employed self-report methods to quantify PA, which are more susceptible to measurement errors compared with the objective measures of PA, such as accelerometers. Accelerometers provide more accurate estimates of PA,^17, 18^ thereby making it easier to disentangle the true associations between PA, adiposity and GS. Moreover, there has been no longitudinal investigation to examine the interplay of adiposity and GS relative to objectively assessed PA. Future PA may be more dependent upon baseline adiposity than strength or vice versa. Hence, it is critical from both epidemiological and public health perspectives to understand the relative contribution of adiposity and GS to PA, in order to promote physically active lifestyles. Therefore, the purpose of this study was to determine the independent and joint longitudinal associations of adiposity and GS with objectively measured PA in middle-aged and older adults participating in a large-scale prospective cohort.

## Methods

### Study design and participants

This study uses data collected from the UK Biobank study, which is an ongoing national cohort study of >500 000 UK adults aged 40–69 years at recruitment. The inclusion criteria included living <25 miles away from one of 22 assessment centres across the UK and being registered with the National Health Service. Each participant underwent baseline measurements between 2006 and 2010 collecting a comprehensive series of genetic, behavioural and biological variables, including measurements of body composition and GS. Several years later (2013–2015), 103 720 of the cohort participated in a sub-study in which their PA was objectively measured by wrist accelerometry.^19^ More detailed descriptions about the rationales and methodology of the UK Biobank project are provided elsewhere.^20^ Each participant signed informed consent before participation. The UK Biobank protocol was approved by the North West Multi-Centre Research Ethics Committee.

### Exposures

#### Grip strength (GS)

GS was assessed once in each hand using a hydraulic hand dynamometer (Jamar J00105, Lafayette, IN, USA), which can measure isometric grip force up to 90 kilograms. The device has good reliability and reproducibility.^21^ Staff calibrated the device at the start of each measurement day. Each participant was asked to sit upright on a chair with their forearm on the armrest, and then grasp the handle of the device in their right hand. They were required to maintain a 90° angle of their elbow adjacent to their side so that their thumb would face upwards, while squeezing the handle as strongly as possible for ~3 s. The same protocol was performed with the left hand. For the current analysis, we used average values of the two hands; unless the value from only one hand was available (*n*=233). Sex- and age-specific quintiles of GS were generated for analyses (Supplementary Table 1).

#### Adiposity measures

Body mass index (BMI) was calculated by dividing measured weight (kg) by measured height squared (m^2^). Waist circumference (WC) was measured by a tape measure at the level of the umbilicus. For the present analyses, BMI (kg m^−^^2^) was categorised as normal weight (18.5–24.9), overweight (25.0–29.9), obesity class I (30.0–34.9) and obesity class II (⩾35.0). A total of 62 men and 373 women who were underweight (BMI<18.5 kg m^−^^2^) were excluded from the analyses because of small sample size. Three categories of WC were generated according to the following sex-specific cut points: ⩽79.9 cm, 80.0–87.9 cm and ⩾88.0 cm for women, and ⩽93.9 cm, 94.0–101.9 cm and ⩾102.0 cm for men.^22^

### Physical activity outcomes

#### Accelerometer data collection

An invitation to participate in the PA sub-study was emailed between February 2013 and December 2015 to 236 519 participants who had given a valid email address upon recruitment. Participants in the North West region who had been involved in other sub-studies were not invited for accelerometer measurement due to the potential participant burden. Enclosed in an envelope, a tri-axial accelerometer (Axivity AX3, Newcastle upon Tyne, UK^23^) configured to capture three-dimensional acceleration at 100 Hz with a dynamic range of ±8 g was mailed, along with instructions on proper use of the device, to consenting participants, who were asked to start wearing the monitor on the dominant wrist (defined as the hand that participants typically use to write) upon receipt and for the next 7 days. Devices were programmed to start and finish recording at pre-specified times. Participants were requested to send the monitor back to the co-ordinating centre in a provided pre-paid envelope at the end of the 7-day monitoring period.

#### Accelerometer data processing

Raw accelerometry data were calibrated to local gravity (1 *g*) with temperature compensation^17^ and filtered to dampen machine noise using a fourth-order Butterworth low-pass filter with a cutoff frequency of 20 Hz. Euclidean Norm Minus One was calculated as the Euclidean Norm (vector magnitude) of the acceleration in three axes minus one gravitational unit (1 *g*), with any negative values truncated to zero. Non-wear was identified as time periods of ⩾60 min where standard deviations of all three axes were <13.0 milli-*g* (1 milli-*g*=0.001 *g*). Overall PA volume (average Euclidean Norm Minus One) and intensity distribution (5-sec resolution) were summarised for each individual activity record, while minimising diurnal bias caused by non-wear.^24^ Moderate-to-vigorous PA (MVPA) was defined as Euclidean Norm Minus One values greater than 125 milli-*g*, and expressed in min day^−1^. Participants with <72 h of wear time or Euclidean Norm Minus One ⩾500 milli-*g* were excluded from the analysis. Previous research found wrist-worn accelerometry to be valid for assessing PA intensity^25^ and energy expenditure.^18, 26^

### Covariates

We included the following variables as covariates in the analysis: age, ethnicity (White, mixed, Asian/Asian British, Black/Black British, others), smoking status (never, previous, current), alcohol consumption (never, previous, currently <3 times/week, currently ⩾3 times/week), employment (unemployed and employed), Townsend Area Deprivation Index (a composite score of employment, car ownership, home ownership and household overcrowding, with higher values indicating a given area’s higher degree of deprivation), severe medical conditions (any of heart attack/stroke/cancer), follow-up time (between baseline assessment and accelerometry measurement), monitor wear time (hours/day), season of accelerometer wear (two orthogonal sine functions), TV time (60-min increments) and baseline self-reported MVPA (min per day). Self-reported MVPA time was calculated as the sum of walking time, non-walking moderate (frequency × duration), and vigorous (frequency × duration) activity time, all of which were reported through questions adapted from the International Physical Activity Questionnaire.^27^

### Statistical analyses

Characteristics of the participants at baseline were summarised by sex and BMI category. Multiple linear regression models were used to investigate the associations of adiposity and GS with the two PA outcomes of average acceleration and MVPA. First, models were fit to estimate associations of adiposity (BMI and WC) with PA, with adjustment for age (Model 1). Remaining aforementioned covariates were additionally adjusted for in subsequent models (Model 2), and finally GS was added (Model 3). A parallel series of models using GS as the primary exposure variable were fit with adjustment for age (Model 1) and additional adjustment for remaining covariates (Model 2), with final adjustment for BMI (Model 3). The GS-activity associations were also estimated, stratified by BMI or WC category, and in parallel the adiposity-activity associations were estimated, stratified by GS quintile (all adjusted for the same covariates as in Model 3); interactions between each exposure and the stratification variable were tested using a multiplication term. All exposure variables were standardised for each sex to facilitate comparison of the strength of association found for different exposures with the same outcome variable. The two PA outcome variables were log-transformed, as the models using untransformed outcome variables did not meet assumptions of normality and heteroscedasticity of residuals. Adjusted means of acceleration and MVPA time for each GS quintile and adiposity category were estimated using marginal effects from the regression analysis. All analyses were performed for men and women, separately. Sensitivity analyses were performed using two alternative thresholds of 100 and 150 milli-*g* for classifying MVPA. Analyses were performed in Stata/SE Version 14 for Windows (StataCorp LP, College Station, TX, USA). Analyses were performed in 2016.

### Code availability

Codes used for processing accelerometer data are provided elsewhere.^28^

## Results

A final sample of 52 161 women and 40 854 men with no missing data for all exposure and outcome variables as well as covariates were included in this study. Table 1 summarises participants’ characteristics across BMI categories for women and men separately. In general, individuals in higher adiposity categories were more likely to be previous or current smokers, have a history of stroke, heart attack or cancer, and report more TV viewing time and less MVPA time at baseline.

Table 2 shows the prospective associations of baseline BMI, WC and GS with average acceleration and MVPA after 5.7 years of follow-up. There were strong inverse associations of BMI and WC with average acceleration and MVPA in both women and men, independent of age (Model 1) plus other potential confounders (Model 2). These associations persisted even after further adjustment for GS (Model 3). GS displayed strong positive associations with average acceleration and MVPA in both women and men, independent of age (Model 1) and other confounders (Model 2). The positive associations remained strong after additional adjustment for BMI (Model 3). In general, the associations for the two adiposity indicators were comparable in size, and stronger than those for GS.

**Figure 1:**
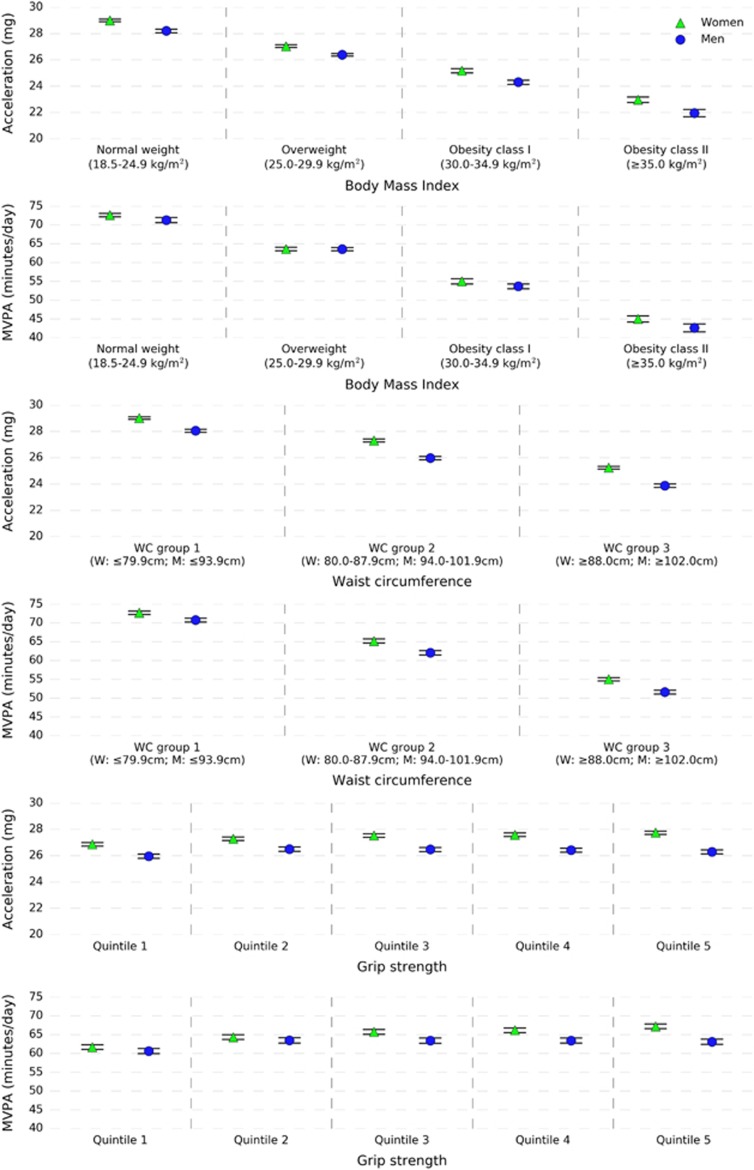
Marginal mean follow-up acceleration levels and moderate-to-vigorous physical activity (MVPA; minutes per day) time across categories of BMI, WC and GS. Marginal means were estimated from linear regression models adjusted for age, ethnicity, smoking status, employment, follow-up period, monitor wear time, season of follow-up assessment, severe medical conditions, TV, baseline self-reported moderate-to-vigorous physical activity time plus GS for BMI, GS for WC or BMI for GS.

Figure 1 shows marginal means of average acceleration and MVPA time for each BMI, WC and GS category for both genders. Average acceleration and MVPA levels were consistently lower across incremental BMI and WC categories in both women and men. Greater quintiles of GS exhibited generally higher levels of average acceleration and MVPA, with the lowest quintile showing consistently the lowest levels.

**Figure 2:**
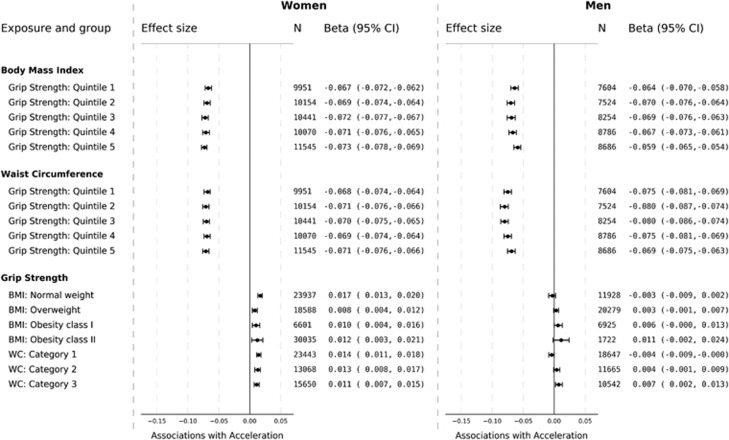
Stratified associations of standardised BMI, WC and GS at baseline with log mean acceleration level after a median 5.7-years follow-up. Models were adjusted for age, ethnicity, smoking status, employment, follow-up period, monitor wear time, seasonality at follow-up, severe medical conditions, TV and baseline self-reported moderate-to-vigorous physical activity time. Values in parentheses indicate 95% confidence intervals. The *P*-values for the interactions between BMI and GS and between WC and GS were 0.063 and 0.494, respectively, in women; 0.212 and <0.0001, respectively, in men. WC categories 1, 2 and 3 were defined as ⩽79.9 cm, 80.0–87.9 cm and ⩾88.0 cm, respectively, for women; ⩽93.9 cm, 94.0–101.9 cm and ⩾102.0 cm, respectively, for men.

Figure 2 shows associations of BMI, WC and GS with average acceleration, stratified by GS quintile. BMI and WC each had strong negative associations with acceleration across all GS strata for both women and men. Strong positive associations between GS and acceleration were observed across all BMI and WC strata in women. However, GS was positively associated with mean acceleration only in obesity class I and WC ⩾102.0 cm in men.

**Figure 3:**
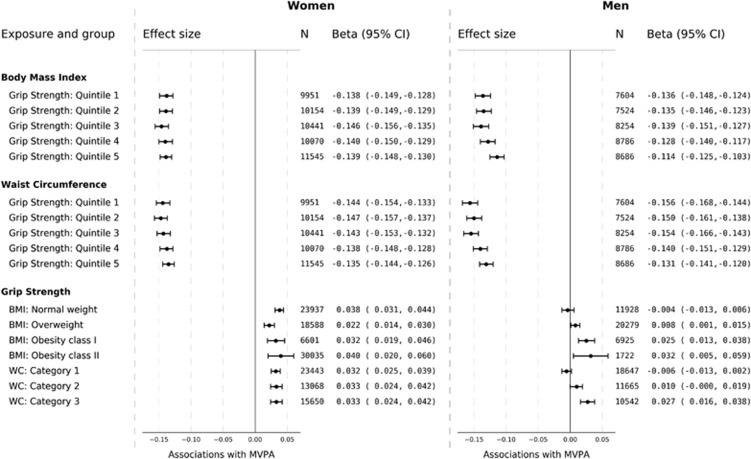
Stratified associations of standardised BMI, WC and GS at baseline with the log of MVPA time after a median 5.7-years follow-up. Models were adjusted for age, ethnicity, smoking status, employment, follow-up period, monitor wear time, seasonality at follow-up, severe medical conditions, TV and baseline self-reported MVPA time. Values in parentheses indicate 95% confidence intervals. The *P*-values for the interactions between BMI and GS and between WC and GS were 0.876 and <0.0001, respectively, in women; 0.003 and <0.0001, respectively, in men. WC categories 1, 2 and 3 were defined as ⩽79.9 cm, 80.0–87.9 cm and ⩾88.0 cm, respectively, for women; ⩽93.9 cm, 94.0–101.9 cm and ⩾102.0 cm, respectively, for men.

Similar patterns of association were identified with MVPA as an outcome variable (Figure 3). For example, strong inverse associations of BMI and WC with MVPA were identified across all GS strata for both women and men. GS showed strong positive associations with MVPA across all BMI and WC strata in women and men, with exceptions for normal weight men and men with WC ⩽93.9 cm.

**Figure 4:**
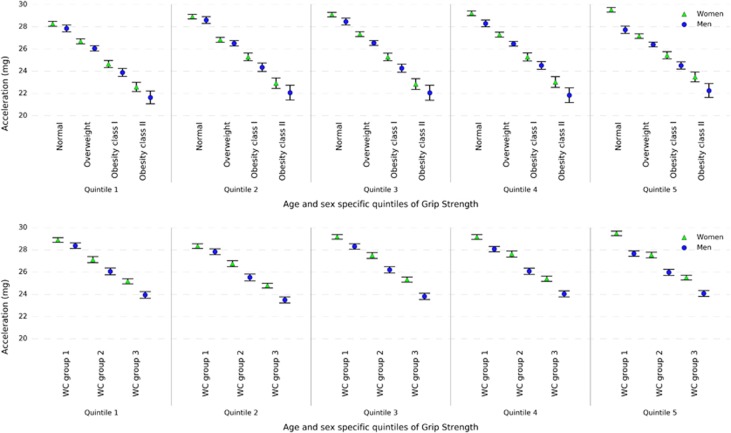
Marginal mean follow-up acceleration levels for each category of BMI and WC across quintiles of GS. Marginal means were estimated from linear regression models (using combined categories of GS and BMI or WC as exposure variables) adjusted for age, ethnicity, smoking status, employment, follow-up period, monitor wear time, season of follow-up assessment, severe medical conditions, TV and baseline self-reported moderate-to-vigorous physical activity time. WC categories 1, 2 and 3 were defined as ⩽79.9 cm, 80.0–87.9 cm and ⩾88.0 cm, respectively, for women; ⩽93.9 cm, 94.0–101.9 cm and ⩾102.0 cm, respectively, for men.

Figure 4 shows marginal means of average acceleration for each BMI and WC category across GS quintiles. In women, average acceleration levels were consistently lower for higher BMI categories across quintiles of GS. Higher GS quintiles also showed higher acceleration levels across BMI categories. For men, consistently lower acceleration levels were observed for higher BMI categories across GS quintiles. There were minimal differences in acceleration between GS quintiles for each BMI category in men, but in general, Quintile 5 had slightly higher acceleration levels compared with the lower GS quintiles in higher BMI categories (that is, overweight, obesity classes I and II). With regards to WC, women in higher WC categories and/or lower GS quintiles had consistently lower acceleration levels. For men, higher WC categories had consistently lower levels of acceleration within each GS quintile. Greater GS quintiles also had minimally higher acceleration levels especially in the two higher WC categories (94–102 cm; >102 cm). Nearly identical patterns of differences were observed with MVPA time as an outcome variable (Supplementary Figure 1). A sensitivity analysis using two alternative thresholds of 100 milli-*g* (Supplementary Figure 2) and 150 milli-*g* for defining MVPA (Supplementary Figure 3) revealed similar trends of associations. The specific values used to create Figures 1 and 4, and Supplementary Figure 1 are presented in Supplementary Tables 2 through 5.

## Discussion

This study is the first to systematically examine the longitudinal associations of adiposity and GS with PA assessed by wrist-worn accelerometry. Overall, baseline BMI, WC and GS all independently predicted subsequent objectively measured PA. In addition, individuals with higher adiposity levels and/or lower GS at baseline tended to have generally lower accelerometer-determined PA levels after a median 5.7-year follow-up, in comparison with those with lower adiposity levels and/or higher GS. Furthermore, higher GS was associated with higher PA levels even in obese individuals. These findings provide compelling justification for PA interventions and/or public health policies to focus on improving body composition and muscle strength in order to increase PA of the population.

The strong negative associations of baseline adiposity with subsequent PA identified herein are consistent with findings from prior longitudinal investigations.^9, 10, 11, 12^ Two early studies found that baseline BMI strongly predicted later self-reported PA characteristics in Danish adult populations, whereas there were no long-term relationships of baseline self-reported PA with follow-up BMI.^11, 12^ Another study concluded that greater weight gain over time was associated with greater probability of being physically inactive 10-years later in English adults.^9^ However, all of those studies^9, 11, 12^ utilised self-reported PA data. A more recent study^10^ using accelerometry found bidirectional longitudinal relationships between various adiposity indices and MVPA (as well as sedentary time) in a small sample of 231 adults with a parental history of type 2 diabetes. The use of raw accelerometry data and a sizable sub-cohort of UK Biobank participants included in the present investigation provides further support for initiating and maintaining public health initiatives to promote active living though improvements in body composition at the population level. Moreover, the examination of adiposity and its interaction with GS relative to accelerometer-derived PA adds considerably to the existing literature.

The positive long-term relationship between GS and PA found in the present study corroborates the recent investigation, which showed baseline GS to predict MVPA at 4.5-year follow-up in a sub-sample of 6599 older adults >60years participating in the UK Biobank study.^16^ However, this study^16^ also used self-reported PA to quantify MVPA, thus subject to potential recall bias. This may have resulted in the weak longitudinal associations between MVPA as an exposure and GS as an outcome. Nonetheless, other longitudinal studies using self-reported PA have found that higher baseline PA was associated with reduced risks of impaired physical function^29, 30, 31, 32^ and sarcopenia,^33^ both of which are related to the age-associated decline of muscle strength. It may be that there are synergistic reciprocal relationships between PA and muscle strength; that is, increased PA through improved muscle strength leads to better mobility, which then results in further increases in PA. Research is clearly needed to further elucidate the bidirectional associations between muscle strength and objectively measured PA.

To our knowledge, this study is the first attempt to explore the interactions of adiposity and strength with PA. In the present study, obese women and men had consistently lower PA levels irrespective of their strength levels. Similarly, less strong women and men had generally lower levels of PA regardless of their weight status, but these associations were less consistent in men with lower levels of BMI and WC. Nevertheless, the relatively higher PA levels with higher GS in the obesity categories suggest that obese individuals may increase PA levels through improvements in muscle strength. Moreover, in overweight/obese individuals, resistance exercise appears to account for greater variability in total energy expenditure than does aerobic exercise.^34^ In addition, previous interventions concluded resistance exercise induced increases in PA levels in young^35^ and older^36^ adults of varying BMI levels. Efforts should, therefore, be placed on improving muscle strength of obese as well as non-obese individuals to increase their PA levels.

There are several strengths of this study. First, all primary exposure (for example, BMI, WC and GS) and outcome variables (for example, acceleration, MVPA) were objectively measured. Objectively measured variables tend to have substantially lower measurement error compared with self-reported methods. Accurate measurement of exposure variables helps to reduce bias in estimated associations, while accurate measurement of outcome variables increases precision.^37^ Second, the use of raw acceleration data collected via wrist-worn accelerometry with documented validity^18^ is a novel aspect of this study. Another strength is the use of data from a large prospective cohort, which enabled comprehensive stratified analyses by multiple categories of BMI, WC and GS in women and men separately.

The following limitations need to be considered when interpreting the findings. First, inference on the causal relationships between adiposity, GS and PA is limited by the observational nature of the study. Moreover, the UK Biobank project did not employ any sampling strategies to recruit a representative sample of UK adults, so findings reported here may not be applicable to the entire UK population, nor adults in other countries. Another potential limitation is the limited evidence on the validity for applying 125 milli-*g* to acceleration data from the dominant wrist to quantify MVPA. However, sensitivity analyses using other cut points (for example, 100 milli-*g* and 150 milli-*g*) revealed almost identical patterns of associations as compared with the results with 125 milli-*g*. Nonetheless, calibration and cross-validation research is needed to develop a wrist methodology that can most accurately assess PA. In addition, the lack of measurements of body composition and GS at follow-up, and accelerometer measurements at baseline in the UK Biobank study prevented investigating the role of change in these characteristics as determinants of objectively measured PA. Another potential limitation is that no further instructions regarding monitor placement were provided to ambidextrous participants who write with both hands, not to left-handed participants who write with their right hand.

## Conclusion

BMI, WC and GS at baseline each independently predicted mean acceleration and MVPA time after a median 5.7-year follow-up. Activity levels at follow-up were lower in more obese individuals regardless of their GS levels; all women and obese men with higher GS had higher future PA levels. Findings of our study provide compelling justification for PA interventions and public health policies to focus on improving body composition and muscle strength, in order to increase PA at the population level.

## Figures and Tables

**Table 1:** Sample characteristics by sex and weight status

	*Women*					*Men*				
	*All (*n=*52 161)*	*Normal weight (*n=*23 937)*	*Overweight (*n=*18 588)*	*Obesity class I (*n=*6601)*	*Obesity class II (*n=*3035)*	*All (*n=*40854)*	*Normal weight (*n=*11 928)*	*Overweight (*n=*20 279)*	*Obesity class I (*n=*6925)*	*Obesity class II (*n=*1722)*
Age	55.6 (7.7)	54.9 (7.8)	56.4 (7.6)	56.1 (7.6)	55.0 (7.6)	56.7 (7.9)	56.2 (8.1)	57.0 (7.9)	57.1 (7.7)	56.1 (7.6)
										
*Ethnicity*										
White	96.8%	97.2%	96.7%	96.2%	95.8%	97.1%	97.1%	97.0%	97.0%	97.6%
Non-White	3.2%	2.8%	3.3%	3.8%	4.2%	2.9%	2.9%	3.0%	3.0%	2.4%
										
*Smoking status*										
Never	60.9%	63.1%	59.6%	58.4%	56.6%	52.3%	60.5%	51.2%	44.0%	42.5%
Previous	33.2%	31.0%	34.6%	35.3%	37.3%	39.5%	31.2%	40.7%	48.0%	49.7%
Current	5.9%	5.9%	5.8%	6.4%	6.1%	8.2%	8.4%	8.1%	8.0%	7.8%
										
*Employment*										
Unemployed	38.9%	36.5%	41.3%	40.9%	38.9%	36.5%	35.5%	36.9%	37.0%	36.6%
Employed	61.1%	63.5%	58.7%	59.2%	61.1%	63.5%	64.5%	63.1%	63.0%	63.4%
										
*Severe medical conditions*										
Any of stroke, heart attack or cancer	9.5%	8.9%	9.6%	10.4%	10.6%	9.5%	8.3%	9.1%	11.8%	13.2%
TV, min per day	146.2 (88.9)	126.8 (81.3)	154.9 (88.0)	174.2 (95.2)	185.2 (98.8)	150.7 (90.5)	127.2 (84.3)	152.6 (87.3)	174.3 (93.6)	195.9 (109.9)
Baseline self-reported MVPA, min per day	84.1 (104.6)	91.9 (109.9)	82.4 (100.9)	72.1 (97.6)	59.1 (88.5)	92.5 (120.0)	95.1 (118.4)	94.6 (121.5)	87.5 (118.5)	69.1 (115.4)
BMI, kg m^−^^2^	26.3 (4.8)	22.6 (1.6)	27.1 (1.4)	32.0 (1.4)	38.9 (3.8)	27.3 (4.0)	23.2 (1.4)	27.2 (1.4)	31.8 (1.4)	38.3 (3.4)
WC, cm	82.8 (11.8)	74.4 (6.1)	84.9 (6.8)	95.7 (7.3)	108.5 (9.7)	95.5 (10.8)	85.5 (6.0)	95.5 (6.1)	106.5 (6.3)	120.7 (9.5)
GS, kg	24.2 (6.1)	24.5 (5.9)	24.1 (6.1)	23.8 (6.4)	23.9 (6.7)	40.3 (8.4)	39.5 (7.9)	40.6 (8.4)	40.6 (9.0)	39.6 (9.3)

Abbreviations: BMI, body mass index; GS, grip strength; IQR, interquartile range; MVPA, moderate-to-vigorous physical activity; WC, waist circumference.

Note: values are means (standard deviations) or proportions.

**Table 2:** Associations of adiposity and grip strength at baseline with follow-up physical activity

*Exposure (per 1-s.d. increment)*	*Outcome (log scale)*	*Women*			*Men*		
		*Model 1*	*Model 2*	*Model 3*	*Model 1*	*Model 2*	*Model 3*
Body mass index	Acceleration	−0.081 (−0.083, −0.078)	−0.071 (−0.073, −0.068)	−0.071 (−0.073, −0.068)	−0.074 (−0.077, −0.072)	−0.066 (−0.069, −0.063)	−0.066 (−0.069, −0.063)
Waist circumference	Acceleration	−0.080 (−0.083, −0.078)	−0.070 (−0.072, −0.068)	−0.070 (−0.072, −0.068)	−0.085 (−0.088, −0.082)	−0.076 (−0.079, −0.073)	−0.076 (−0.079, −0.073)
Grip strength	Acceleration	0.018 (0.015, 0.020)	0.013 (0.010, 0.015)	0.012 (0.010, 0.015)	0.002 (−0.0004, 0.005)	0.0004 (−0.002, 0.003)	0.003 (0.001, 0.006)
Body mass index	MVPA	−0.156 (−0.161, −0.152)	−0.141 (−0.145, −0.136)	−0.141 (−0.145, −0.136)	−0.143 (−0.148, −0.138)	−0.131 (−0.136, −0.125)	−0.131 (−0.136, −0.126)
Waist circumference	MVPA	−0.158 (−0.162, −0.153)	−0.141(−0.146, −0.137)	−0.142 (−0.146, −0.137)	−0.161 (−0.166 −0.156)	−0.147 (−0.152 −0.142)	−0.147 (−0.152, −0.142)
Grip strength	MVPA	0.041 (0.036, 0.046)	0.033 (0.028, 0.037)	0.032 (0.028, 0.037)	0.010 (0.004, 0.015)	0.007 (0.001, 0.012)	0.012 (0.007, 0.017)

Abbreviation: MPVA, moderate-to-vigorous physical activity.

Model 1 adjusted for age.

Model 2 adjusted for age, ethnicity, smoking status, employment, follow-up time, monitor wear time, seasonality at follow-up, severe medical conditions, TV and baseline self-reported MVPA time.

Model 3 adjusted for all covariates included in Model 2 and grip strength when body mass index or waist circumference was the exposure, or body mass index when grip strength was the exposure.

Note: the *β*-coefficients and 95% confidence intervals (values in parentheses) are reported on a log scale.
