# Supplementary material for: Adiposity and grip strength as long-term predictors of objectively measured physical activity in 93 015 adults: the UK Biobank study
Source: Int J Obes (Lond). 2017 Jun 6;41(9):1361–8. doi: 10.1038/ijo.2017.122 (PMC5578433; doi:10.1038/ijo.2017.122)
Supplement: Supplementary Material [file ijo2017122x1.docx]

Supplementary Table 1. Age- and sex-specific cut-points used to create quintiles of grip strength

| Age range | Quintiles of grip strength | Men (kg) | Women (kg) |
| --- | --- | --- | --- |
| <50yrs | Q1 | <36.0 | <22.0 |
|  | Q2 | 36.0-40.9 | 22.0-25.9 |
|  | Q3 | 41.0-44.9 | 26.0-28.9 |
|  | Q4 | 45.0-49.9 | 29.0-31.9 |
|  | Q5 | ≥50.0 | ≥32.0 |
|  |  |  |  |
| 50-59yrs | Q1 | <34.0 | <20.0 |
|  | Q2 | 34.0-38.9 | 20.0-22.9 |
|  | Q3 | 39.0-42.9 | 23.0-25.9 |
|  | Q4 | 43.0-47.9 | 26.0-28.9 |
|  | Q5 | ≥48.0 | ≥29.0 |
|  |  |  |  |
| ≥60yrs | Q1 | <32.0 | <18.0 |
|  | Q2 | 32.0-35.9 | 18.0-20.9 |
|  | Q3 | 36.0-39.9 | 21.0-23.4 |
|  | Q4 | 40.0-44.4 | 23.5-26.9 |
|  | Q5 | ≥44.5 | ≥27.0 |

Supplementary Table 2. Marginal means of follow-up acceleration levels (milli-*g*)

| Exposure |  | Women |  |  | Men |  |  |
| --- | --- | --- | --- | --- | --- | --- | --- |
| Type | Category | Mean | 95% CI – Lower bound | 95% CI – Upper bound | Mean | 95% CI – Lower bound | 95% CI – Upper bound |
| Body mass index |  |  |  |  |  |  |  |
|  | Normal weight  (18.5-24.9 kg/m^2^) | 29.0 | 28.9 | 29.1 | 28.2 | 28.1 | 28.4 |
|  | Overweight  (25.0-29.9 kg/m^2^) | 27.1 | 27.0 | 27.2 | 26.4 | 26.3 | 26.5 |
|  | Obesity class I  (30.0-34.9 kg/m^2^) | 25.2 | 25.0 | 25.3 | 24.3 | 24.2 | 24.5 |
|  | Obesity class II  (≥35.0 kg/m^2^) | 23.0 | 22.8 | 23.2 | 22.0 | 21.7 | 22.2 |
| Waist circumference |  |  |  |  |  |  |  |
|  | ≤79.9cm (W) or <94.0cm (M) | 29.0 | 28.9 | 29.1 | 28.1 | 28.0 | 28.2 |
|  | 80.0-87.9cm (W) or 94.0-101.9cm (M) | 27.3 | 27.2 | 27.4 | 26.0 | 25.9 | 26.1 |
|  | ≥88.0cm (W), or ≥102.0cm (M) | 25.3 | 25.2 | 25.4 | 23.9 | 23.8 | 24.0 |
| Grip strength |  |  |  |  |  |  |  |
|  | Quintile 1 | 26.9 | 26.7 | 27.0 | 26.0 | 25.8 | 26.1 |
|  | Quintile 2 | 27.3 | 27.2 | 27.4 | 26.5 | 26.3 | 26.7 |
|  | Quintile 3 | 27.5 | 27.4 | 27.7 | 26.5 | 26.3 | 26.6 |
|  | Quintile 4 | 27.6 | 27.5 | 27.7 | 26.4 | 26.3 | 26.6 |
|  | Quintile 5 | 27.8 | 27.6 | 27.9 | 26.3 | 26.2 | 26.5 |

Note: “W” for women and M for “Men”

Supplementary Table 3. Marginal means of follow-up moderate-to-vigorous physical activity (MVPA; minutes/day) levels using a cut-point of 125milli-*g*

| Exposure |  | Women |  |  | Men |  |  |
| --- | --- | --- | --- | --- | --- | --- | --- |
| Type | Category | Mean | 95% CI – Lower bound | 95% CI – Upper bound | Mean | 95% CI – Lower bound | 95% CI – Upper bound |
| Body mass index |  |  |  |  |  |  |  |
|  | Normal weight  (18.5-24.9 kg/m^2^) | 72.7 | 72.2 | 73.1 | 71.3 | 70.7 | 72.0 |
|  | Overweight  (25.0-29.9 kg/m^2^) | 63.7 | 63.2 | 64.1 | 63.6 | 63.2 | 64.1 |
|  | Obesity class I  (30.0-34.9 kg/m^2^) | 55.0 | 54.4 | 55.7 | 53.7 | 53.1 | 54.4 |
|  | Obesity class II  (≥35.0 kg/m^2^) | 45.0 | 44.2 | 45.8 | 42.6 | 41.6 | 43.7 |
| Waist circumference |  |  |  |  |  |  |  |
|  | ≤79.9cm (W) or <94.0cm (M) | 72.7 | 72.3 | 73.2 | 70.8 | 70.3 | 71.3 |
|  | 80.0-87.9cm (W) or 94.0-101.9cm (M) | 65.2 | 64.6 | 65.8 | 62.1 | 61.5 | 62.7 |
|  | ≥88.0cm (W), or ≥102.0cm (M) | 55.0 | 54.6 | 55.5 | 51.6 | 51.1 | 52.1 |
| Grip strength |  |  |  |  |  |  |  |
|  | Quintile 1 | 61.7 | 61.1 | 62.3 | 60.7 | 60.0 | 61.4 |
|  | Quintile 2 | 64.4 | 63.7 | 65.0 | 63.5 | 62.8 | 64.2 |
|  | Quintile 3 | 65.8 | 65.1 | 66.4 | 63.4 | 62.7 | 64.1 |
|  | Quintile 4 | 66.2 | 65.6 | 66.8 | 63.4 | 62.8 | 64.1 |
|  | Quintile 5 | 67.2 | 66.6 | 67.8 | 63.1 | 62.4 | 63.8 |

Note: “W” for women and M for “Men”

Supplementary Table 4. Marginal means of follow-up acceleration levels (milli-*g*) across combined categories of adiposity and grip strength

| Adiposity |  | Grip strength | Women |  |  | Men |  |  |
| --- | --- | --- | --- | --- | --- | --- | --- | --- |
| Type | Category |  | Mean | 95% CI – Lower bound | 95% CI – Upper bound | Mean | 95% CI – Lower bound | 95% CI – Upper bound |
| Body mass index |  |  |  |  |  |  |  |  |
|  | Normal weight | Quintile 1 | 29.5 | 29.3 | 29.7 | 27.7 | 27.4 | 28.1 |
|  | Overweight |  | 27.2 | 26.9 | 27.4 | 26.4 | 26.2 | 26.6 |
|  | Obesity class I |  | 25.4 | 25.1 | 25.8 | 24.5 | 24.2 | 24.8 |
|  | Obesity class II |  | 23.5 | 23.0 | 23.9 | 22.2 | 21.6 | 22.9 |
|  | Normal weight | Quintile 2 | 29.2 | 29.0 | 29.4 | 28.3 | 28.0 | 28.6 |
|  | Overweight |  | 27.3 | 27.1 | 27.5 | 26.5 | 26.3 | 26.7 |
|  | Obesity class I |  | 25.3 | 24.9 | 25.6 | 24.5 | 24.2 | 24.9 |
|  | Obesity class II |  | 23.0 | 22.5 | 23.5 | 21.8 | 21.2 | 22.5 |
|  | Normal weight | Quintile 3 | 29.1 | 28.9 | 29.3 | 28.5 | 28.2 | 28.8 |
|  | Overweight |  | 27.3 | 27.1 | 27.6 | 26.5 | 26.3 | 26.8 |
|  | Obesity class I |  | 25.3 | 24.9 | 25.6 | 24.3 | 23.9 | 24.6 |
|  | Obesity class II |  | 22.8 | 22.4 | 23.3 | 22.1 | 21.4 | 22.7 |
|  | Normal weight | Quintile 4 | 28.9 | 28.7 | 29.1 | 28.6 | 28.3 | 28.9 |
|  | Overweight |  | 26.8 | 26.6 | 27.0 | 26.5 | 26.3 | 26.8 |
|  | Obesity class I |  | 25.3 | 24.9 | 25.6 | 24.4 | 24.0 | 24.7 |
|  | Obesity class II |  | 22.9 | 22.5 | 23.4 | 22.1 | 21.4 | 22.7 |
|  | Normal weight | Quintile 5 | 28.3 | 28.0 | 28.5 | 27.8 | 27.5 | 28.1 |
|  | Overweight |  | 26.7 | 26.5 | 26.9 | 26.1 | 25.8 | 26.3 |
|  | Obesity class I |  | 24.6 | 24.3 | 25.0 | 23.9 | 23.5 | 24.2 |
|  | Obesity class II |  | 22.6 | 22.2 | 23.0 | 21.6 | 21.1 | 22.2 |
| Waist circumference |  |  |  |  |  |  |  |  |
|  | ≤79.9cm (W) or <94.0cm (M) | Quintile 1 | 29.5 | 29.3 | 29.7 | 27.7 | 27.4 | 27.9 |
|  | 80.0-87.9cm (W) or 94.0-101.9cm (M) |  | 27.5 | 27.3 | 27.8 | 26.0 | 25.7 | 26.3 |
|  | ≥88.0cm (W), or ≥102.0cm (M) |  | 25.5 | 25.3 | 25.7 | 24.1 | 23.8 | 24.4 |
|  | ≤79.9cm (W) or <94.0cm (M) | Quintile 2 | 29.2 | 29.0 | 29.4 | 28.1 | 27.8 | 28.3 |
|  | 80.0-87.9cm (W) or 94.0-101.9cm (M) |  | 27.6 | 27.4 | 27.9 | 26.1 | 25.8 | 26.4 |
|  | ≥88.0cm (W), or ≥102.0cm (M) |  | 25.4 | 25.2 | 25.6 | 24.0 | 23.8 | 24.3 |
|  | ≤79.9cm (W) or <94.0cm (M) | Quintile 3 | 29.2 | 29.0 | 29.4 | 28.3 | 28.1 | 28.6 |
|  | 80.0-87.9cm (W) or 94.0-101.9cm (M) |  | 27.5 | 27.2 | 27.8 | 26.2 | 25.9 | 26.5 |
|  | ≥88.0cm (W), or ≥102.0cm (M) |  | 25.3 | 25.1 | 25.6 | 23.8 | 23.5 | 24.1 |
|  | ≤79.9cm (W) or <94.0cm (M) | Quintile 4 | 28.9 | 28.7 | 29.1 | 28.4 | 28.1 | 28.6 |
|  | 80.0-87.9cm (W) or 94.0-101.9cm (M) |  | 27.1 | 26.9 | 27.4 | 26.1 | 25.8 | 26.4 |
|  | ≥88.0cm (W), or ≥102.0cm (M) |  | 25.2 | 25.0 | 25.4 | 24.0 | 23.7 | 24.3 |
|  | ≤79.9cm (W) or <94.0cm (M) | Quintile 5 | 28.4 | 28.1 | 28.6 | 27.8 | 27.6 | 28.1 |
|  | 80.0-87.9cm (W) or 94.0-101.9cm (M) |  | 26.8 | 26.5 | 27.0 | 25.5 | 25.2 | 25.8 |
|  | ≥88.0cm (W), or ≥102.0cm (M) |  | 24.8 | 24.6 | 25.0 | 23.5 | 23.2 | 23.8 |

Note: “W” for women and M for “Men”

Supplementary Table 5. Marginal means of follow-up moderate-to-vigorous physical activity (MVPA; minutes/day) levels using a cut-point of 125milli-*g* across combined categories of adiposity and grip strength.

| Adiposity |  | Grip strength | Women |  |  | Men |  |  |
| --- | --- | --- | --- | --- | --- | --- | --- | --- |
| Type | Category |  | Mean | 95% CI – Lower bound | 95% CI – Upper bound | Mean | 95% CI – Lower bound | 95% CI – Upper bound |
| Body mass index |  |  |  |  |  |  |  |  |
|  | Normal weight | Quintile 1 | 75.4 | 74.4 | 76.5 | 69.3 | 67.7 | 70.8 |
|  | Overweight |  | 64.7 | 63.7 | 65.6 | 63.7 | 62.8 | 64.7 |
|  | Obesity class I |  | 57.2 | 55.7 | 58.6 | 55.3 | 54.0 | 56.7 |
|  | Obesity class II |  | 48.1 | 46.4 | 50.0 | 44.8 | 42.5 | 47.2 |
|  | Normal weight | Quintile 2 | 73.7 | 72.7 | 74.8 | 71.9 | 70.5 | 73.4 |
|  | Overweight |  | 65.0 | 64.0 | 66.1 | 63.8 | 62.9 | 64.8 |
|  | Obesity class I |  | 56.0 | 54.5 | 57.7 | 55.3 | 53.9 | 56.8 |
|  | Obesity class II |  | 45.5 | 43.6 | 47.4 | 42.3 | 40.0 | 44.7 |
|  | Normal weight | Quintile 3 | 73.1 | 72.1 | 74.2 | 72.3 | 70.9 | 73.8 |
|  | Overweight |  | 65.2 | 64.2 | 66.3 | 64.4 | 63.4 | 65.4 |
|  | Obesity class I |  | 55.3 | 53.8 | 56.8 | 53.1 | 51.7 | 54.6 |
|  | Obesity class II |  | 44.2 | 42.4 | 46.1 | 43.0 | 40.6 | 45.5 |
|  | Normal weight | Quintile 4 | 72.1 | 71.1 | 73.2 | 72.4 | 71.0 | 73.9 |
|  | Overweight |  | 62.4 | 61.4 | 63.4 | 64.5 | 63.4 | 65.5 |
|  | Obesity class I |  | 55.1 | 53.7 | 56.7 | 54.0 | 52.5 | 55.6 |
|  | Obesity class II |  | 44.6 | 42.8 | 46.5 | 43.0 | 40.7 | 45.5 |
|  | Normal weight | Quintile 5 | 68.5 | 67.5 | 69.6 | 70.0 | 68.6 | 71.5 |
|  | Overweight |  | 61.0 | 60.0 | 62.0 | 61.6 | 60.6 | 62.7 |
|  | Obesity class I |  | 51.6 | 50.3 | 53.0 | 50.4 | 49.0 | 51.8 |
|  | Obesity class II |  | 42.4 | 40.9 | 44.0 | 40.4 | 38.5 | 42.5 |
| Waist circumference |  |  |  |  |  |  |  |  |
|  | ≤79.9cm (W) or <94.0cm (M) | Quintile 1 | 75.0 | 74.0 | 76.1 | 69.1 | 68.0 | 70.2 |
|  | 80.0-87.9cm (W) or 94.0-101.9cm (M) |  | 66.8 | 65.6 | 68.0 | 62.4 | 61.2 | 63.6 |
|  | ≥88.0cm (W), or ≥102.0cm (M) |  | 57.3 | 56.3 | 58.2 | 53.1 | 52.0 | 54.2 |
|  | ≤79.9cm (W) or <94.0cm (M) | Quintile 2 | 73.6 | 72.6 | 74.7 | 71.0 | 69.9 | 72.1 |
|  | 80.0-87.9cm (W) or 94.0-101.9cm (M) |  | 66.7 | 65.4 | 68.0 | 62.3 | 61.0 | 63.5 |
|  | ≥88.0cm (W), or ≥102.0cm (M) |  | 56.2 | 55.2 | 57.3 | 53.0 | 51.9 | 54.1 |
|  | ≤79.9cm (W) or <94.0cm (M) | Quintile 3 | 73.5 | 72.5 | 74.6 | 72.0 | 70.8 | 73.2 |
|  | 80.0-87.9cm (W) or 94.0-101.9cm (M) |  | 66.5 | 65.3 | 67.8 | 62.9 | 61.7 | 64.2 |
|  | ≥88.0cm (W), or ≥102.0cm (M) |  | 55.2 | 54.2 | 56.2 | 51.4 | 50.2 | 52.5 |
|  | ≤79.9cm (W) or <94.0cm (M) | Quintile 4 | 72.0 | 71.0 | 73.0 | 71.8 | 70.6 | 73.0 |
|  | 80.0-87.9cm (W) or 94.0-101.9cm (M) |  | 64.3 | 63.0 | 65.6 | 63.0 | 61.6 | 64.4 |
|  | ≥88.0cm (W), or ≥102.0cm (M) |  | 54.5 | 53.5 | 55.5 | 51.9 | 50.7 | 53.2 |
|  | ≤79.9cm (W) or <94.0cm (M) | Quintile 5 | 69.2 | 68.1 | 70.2 | 69.9 | 68.8 | 71.1 |
|  | 80.0-87.9cm (W) or 94.0-101.9cm (M) |  | 61.6 | 60.4 | 62.9 | 59.7 | 58.4 | 61.1 |
|  | ≥88.0cm (W), or ≥102.0cm (M) |  | 51.9 | 51.0 | 52.8 | 48.6 | 47.6 | 49.7 |

Note: “W” for women and M for “Men”


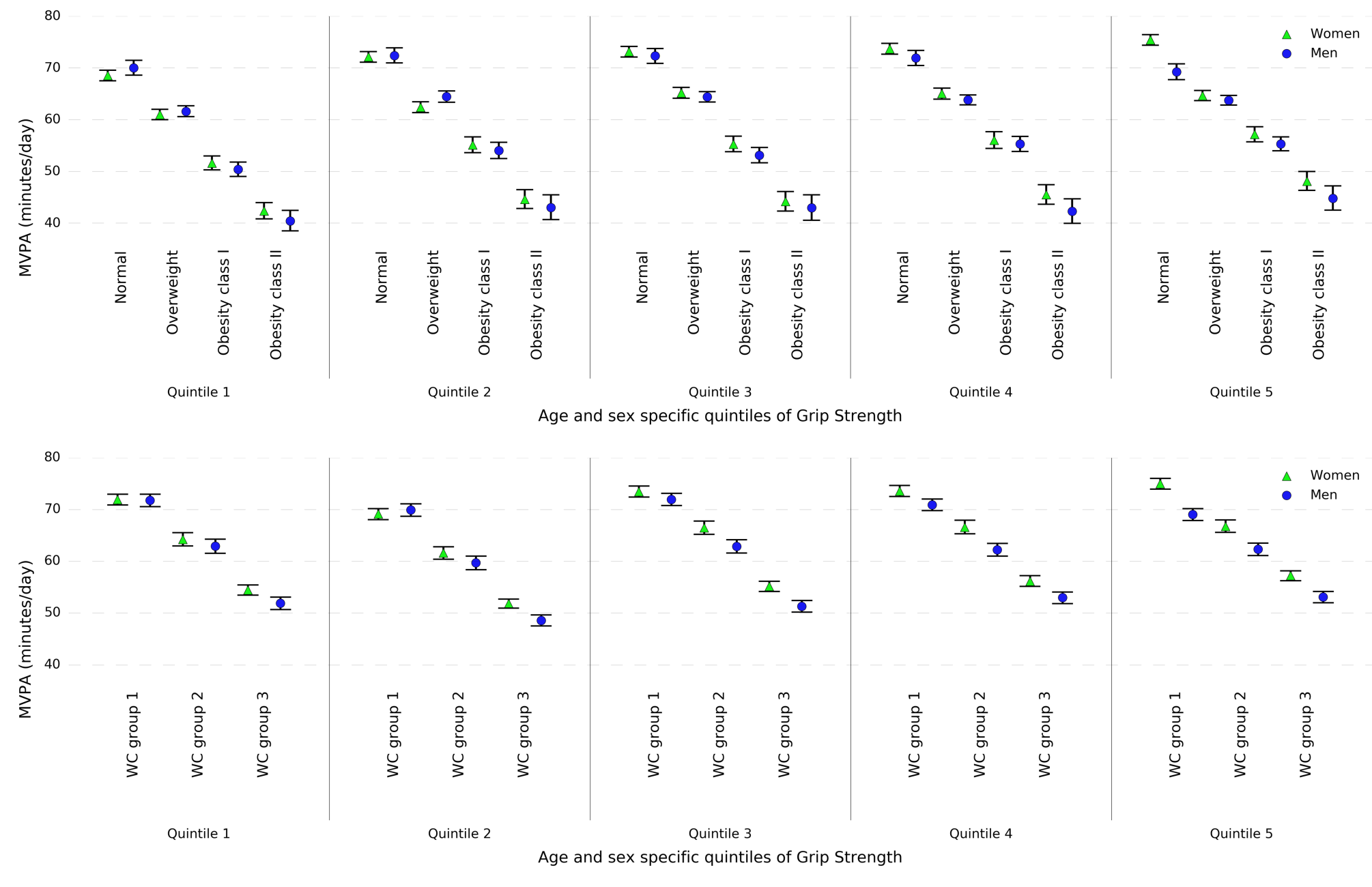


Supplementary Figure 1. Marginal mean follow-up moderate-to-vigorous physical activity (MVPA; minutes/day) time for each category of baseline body mass index (BMI) and waist circumference (WC) across quintiles of grip strength (GS). Note: Marginal means were obtained from linear regression models (using combined categories of grip strength and body mass index or waist circumference as exposure variables) adjusted for age, ethnicity, smoking status, employment, follow-up period, monitor wear time, season of follow-up assessment, severe medical conditions, TV and baseline self-reported MVPA time. WC categories 1, 2, and 3 were defined as ≤79.9cm, 80.0-87.9cm and ≥88.0cm, respectively, for women; <93.9cm, 94.0-101.9cm and ≥102.0cm, respectively, for men.


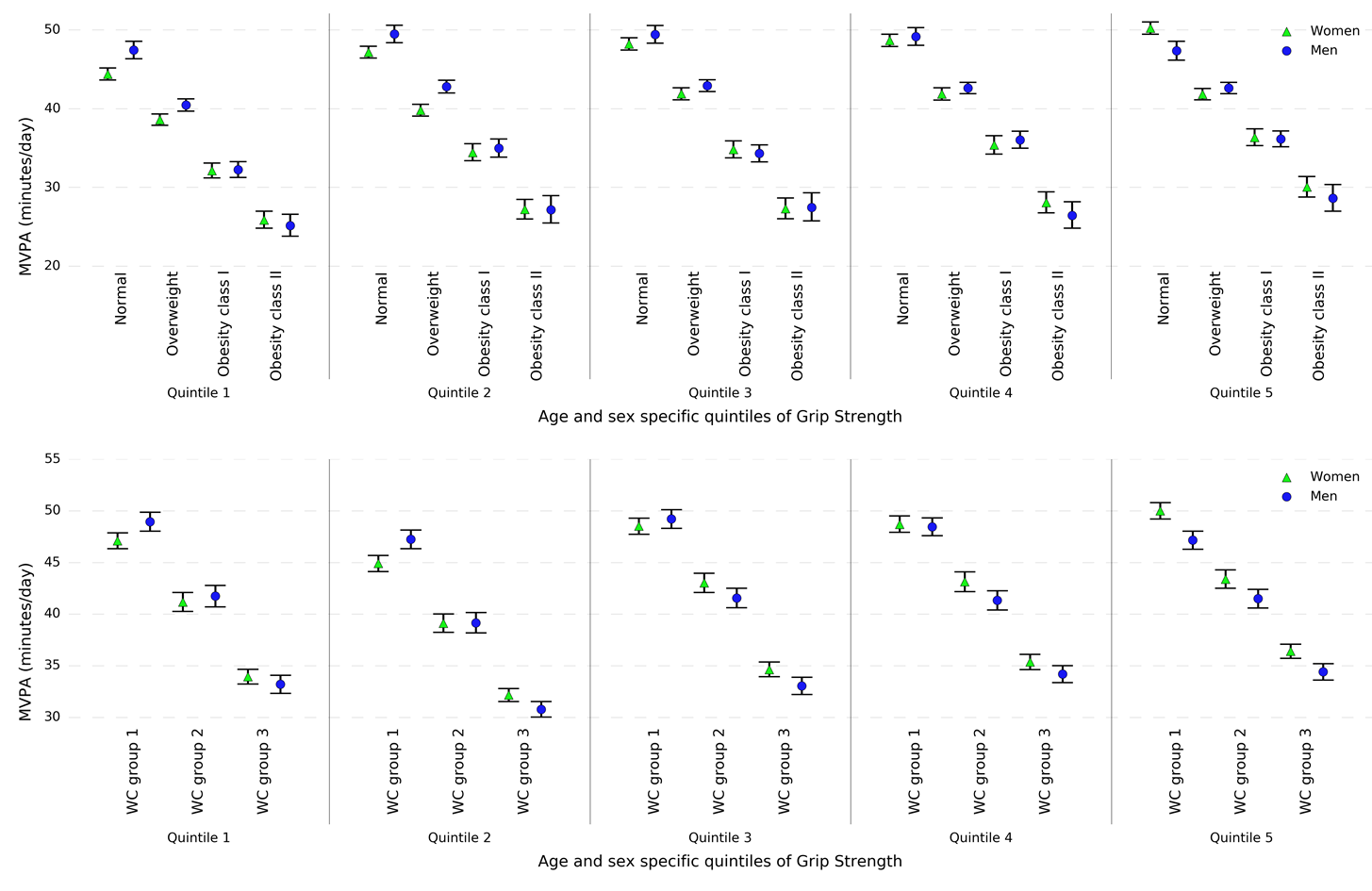


Supplementary Figure 2. Marginal means of follow-up moderate-to-vigorous physical activity (MVPA; minutes/day) time estimated with an alternative cut-point of 100milli-*g* for each category of body mass index (BMI; top) and waist circumference (WC; bottom) across quintiles of grip strength (GS). Note: Marginal means were obtained from linear regression models (using combined categories of grip strength and body mass index or waist circumference as exposure variables) adjusted for age, ethnicity, smoking status, employment, follow-up period, monitor wear time, seasonality at follow-up, severe medical conditions, TV and baseline self-reported MVPA time. WC categories 1, 2, and 3 were defined as ≤79.9cm, 80.0-87.9cm and ≥88.0cm, respectively, for women; <94.0cm, 94.0-101.9cm and ≥102.0cm, respectively, for men.


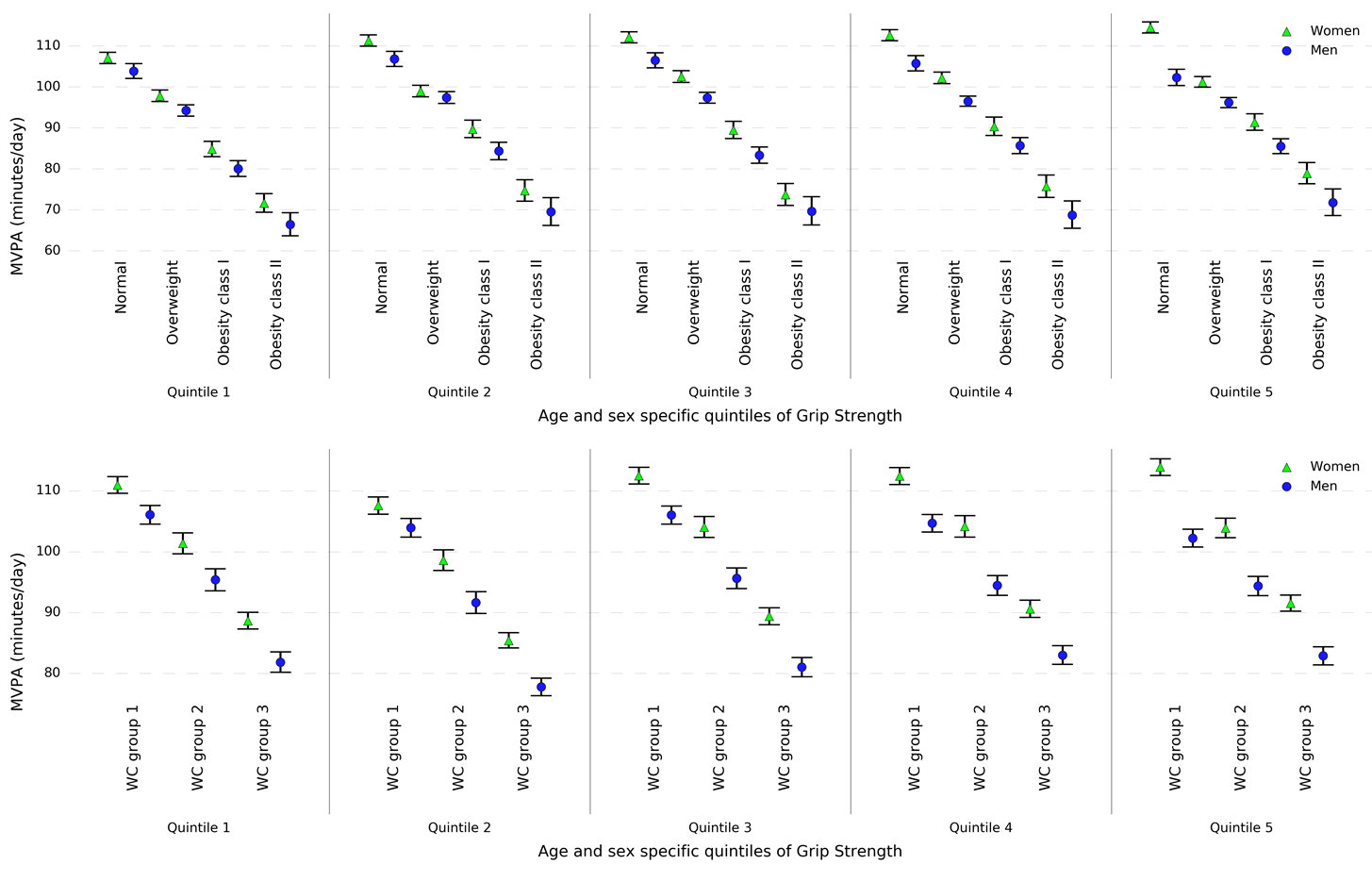


Supplementary Figure 3. Marginal means of follow-up moderate-to-vigorous physical activity (MVPA; minutes/day) time estimated with an alternative cut-point of 150milli-*g* for each category of body mass index (BMI; top) and waist circumference (WC; bottom) across quintiles of grip strength (GS). Note: Marginal means were obtained from linear regression models (using combined categories of grip strength and body mass index or waist circumference as exposure variables) adjusted for age, ethnicity, smoking status, employment, follow-up period, monitor wear time, seasonality at follow-up, severe medical conditions, TV and baseline self-reported MVPA time. WC categories 1, 2, and 3 were defined as ≤79.9cm, 80.0-87.9cm and ≥88.0cm, respectively, for women; <94.0cm, 94.0-101.9cm and ≥102.0cm, respectively, for men.
